# Métastases choroïdiennes bilatérales d'origine inconnue: à propos d'un cas

**DOI:** 10.11604/pamj.2014.19.350.5249

**Published:** 2014-12-03

**Authors:** Fadoua Alami, Imane Ahmiti, Ramzia Sebbah, Mohamed El Yadari, Bahia Ouazzani, Amina Berraho

**Affiliations:** 1 Service d'Ophtalmologie B, Hôpital des Spécialités, Rabat, Maroc

**Keywords:** Métamorphopsies, métastases, choroïde, metamorphopsia, metastases, choroid

## Abstract

Nous rapportons l'observation d'un patient de 52 ans sans antécédents pathologiques notables, qui présente depuis 10 mois un flou visuel de l’œil droit et des métamorphopsies au niveau de l’œil gauche, suivies de troubles visuels très gênants et baisse importante de l'acuité visuel du coté droit, motivant une consultation. Le bilan oculaire a mis en évidence des métastases choroïdiennes bilatérales; le bilan d'extension révèle des métastases multifocales dont le site primitif est inconnu, notre patient a décédé durant les jours d'explorations.

## Introduction

Les métastases choroïdiennes sont des tumeurs malignes oculaires plus fréquentes que les mélanomes choroïdiens [1], qui apparaissant généralement dans un contexte défavorable de cancer multi-métastasé [2]; leur fréquence reste sous estimée par l'absence de leur recherche systématique; d'après les dernières publications, elle est de l'ordre de 10 à 38% [1] Près d'un tiers des patients atteints de métastases choroïdiennes n'ont pas d'antécédents de cancer et l'ophtalmologiste peut être le premier médecin à détecter la tumeur primaire [3, 4]. A travers cette observation clinique, les auteurs rappellent les caractéristiques cliniques et échographiques des métastases choroïdiennes afin de souligner le rôle de l'ophtalmologiste dans la conduite de l'exploration pour trouver l'origine occulte primaire et guider la prise en charge thérapeutique, seul garant d'un meilleur pronostic.

## Méthodes

Nous rapportons le cas d'un patient de 52 ans, sans aucun antécédent médico-chirurgical et pas de notion de tabagisme, qui se présente en consultation ophtalmologique du CHU de Rabat pour un flou visuel de l’œil droit depuis 10 mois et des métamorphopsies au niveau de l’œil gauche, suivies de troubles visuels très gênants et baisse importante de l'acuité visuel du coté droit, le patient a bénéficié d'un examen ophtalmologique complet et un bilan radiologique et biologique en fonction des orientations cliniques.

## Résultats

**Figure 1:**
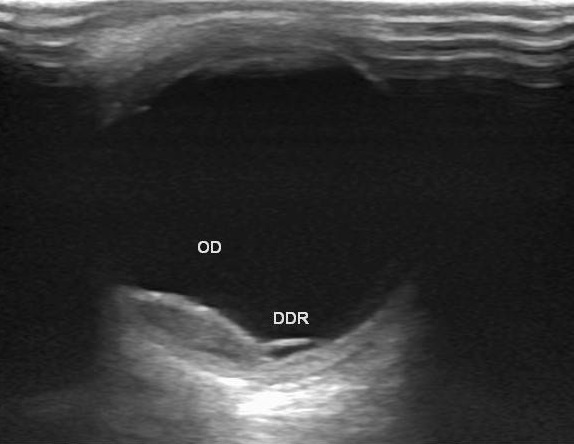
Échographie oculaire en mode B de l’œil droit montre une masse inféro-nasal d'aspect tissulaire, hypo-écho gène, adhérente à la choroïde mesurant 11mm/3 mm avec décollement séreux rétinien sans excavation choroïdienne

**Figure 2:**
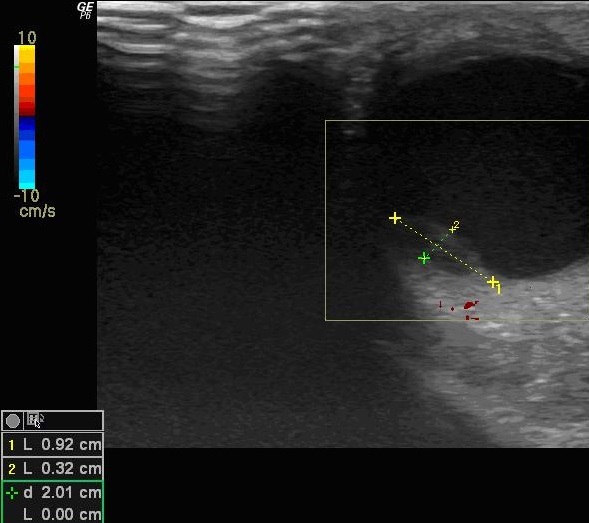
Échographie oculaire en mode B de l’œil gauche montre une masse choroïdienne temporale mesurant 9mm/3mm à contenu tissulaire hypo-écho gene

**Figure 3:**
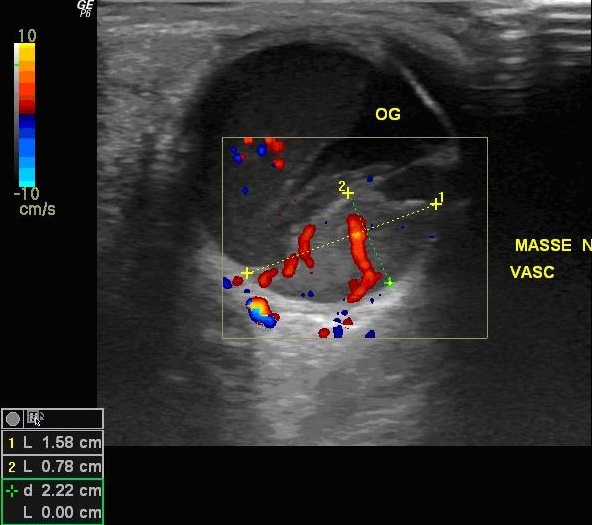
Échographie oculaire en mode B couplée au doppler montre une volumineuse masse nasale 15mm/ 7,8 mm, à surface irrégulière à contenu tissulaire hétérogène présentant une vascularisation centrale évidente au doppler avec soulèvement rétinien et épanchement sous rétinien

L'examen ophtalmologique trouve une acuité visuelle à MDD de l’œil droit et 3/10 l’œil gauche inaméliorable. L'examen du segment antérieur note à gauche une hyperhémie conjonctivale, une hyalite, le segment antérieur de l’œil droit est sans anomalies. Le tonus oculaire est correct en ODG. L'examen du fond d’œil révèle la présence, à droite d'une lésion blanc jaunâtre nasale, non pigmentée, à bords flous, la papille est normale et la rétine est à plat, à gauche, on note que l'examen est difficile vu l’état du segment antérieur. L’échographie oculaire retrouve à droite une masse d'aspect tissulaire hypo-échogène adhérente à la choroïde inféro-nasal mesurant 11/ 3 mm, avec soulèvement de la rétine (Figure 1), à gauche on note l'association de deux masses sous-rétiniennes temporal de 9 / 3 mm (Figure 2) et nasal de 15,8/ 7,8mm (Figure 3) avec décollements séreux rétiniens et l'absence d'excavation choroïdienne en ODG. Le doppler met en évidence une vascularisation centrale des masses choroïdiennes (Figure 3) Devant ce tableau, on évoque des métastases intraoculaires, un mélanome choroïdien ou un hémangiome choroïdien. L'aspect ophtalmoscopique et échographique est plus en faveur de métastases choroïdiennes bilatérales plus marqué à gauche. Un bilan général est débuté en urgence pour la recherche du site primitif. L'examen général trouve un patient en mauvais état général sans adénopathie à l'examen des aires ganglionnaire et pas de lésion cutanée suspecte Le bilan biologique NFS, VS, Ionogramme, bilan hépatique est normal La recherche des marqueurs tumoraux (CA 15,3) s'est révélée négative. Un bilan radiologique (radio pulmonaire, scanner cérébral et thoracique, échographie abdomino-pelvienne, scintigraphie osseuse) est fait: Le scanner thoracique montre un processus lésionnel nodulaire du lobe inférieur pulmonaire gauche accolé au péricarde, une coulée d'adénomégalie du hile pulmonaire gauche avec un discret épanchement pleural gauche Le scanner abdominal montre de multiples lésions évocatrices de métastases hépatiques sans aucune lésion des autres viscères. Les résultats des ponctions, biopsies hépatique et pulmonaire, révèlent un aspect cytologique de métastases d'un adénocarcinome mais aucune preuve de la tumeur primaire. Le scanner cérébral n'a mis en évidence aucune lésion. La fibroscopie gastrique s'est révélée normale, tout comme la coloscopie et la scintigraphie thyroïdienne. Durant les jours des explorations paracliniques, le patient a accusé une altération importante de l’état générale est a décédé par un choc septique en réanimation, l'autopsie était proposée mais refusée par la famille.

## Discussion

On retient de notre observation que les métastases de la choroïde peuvent se voir dans un contexte de métastases multifocales dont la néoplasie primitive reste inconnue malgré une enquête approfondie du système pour rechercher le site principal probable. Le cas rapporté est original du fait de la faible fréquence des métastases choroïdiennes bilatérales et de leur caractère multifocale surtout quand la néoplasie primitive est inconnue Au travers de la littérature, dans 20% des cas, la tumeur primitive est inconnue au moment du diagnostic de métastase orbitaire [5]. Shields et al [4] dans leur expérience de 520 yeux avec métastase choroïdienne, a noté que 34% des patients n'a pas d'antécédents de cancer. Dans environ la moitié d'entre eux (17%), le site primaire est resté non diagnostiqué en dépit de vastes enquêtes systémiques. Des expériences similaires ont été trouvées dans d'autres séries de ces patients [6, 7]. Le bilan d'extension doit être le plus large possible, afin de trouver l'origine du primitif; Il devrait inclure une formule sanguine complète, un bilan hépatique et rénal, tests de la fonction thyroïdienne et les marqueurs tumoraux. Un bilan radiologique complet avec radiographie pulmonaire, échographie abdominale et pelvienne, et une mammographie. Un ensemble de balayage corps de CT et scintigraphie osseuse doit être ordonné [8]. Il faut souligner l'intérêt des fibroscopies, biopsies, même en l'absence de lésions radio-scannographiques [1]. Le traitement fera appel simultanément à la radiothérapie, à la chimiothérapie et à l'hormonothérapie [1]. Le pronostic reste sombre, 85% des patients meurent dans l'année du diagnostic et seuls -10% d'entre eux peut être survivants à long terme [8] Dans notre travail, la biopsie hépatique et pulmonaire révèle un aspect cytologique de métastases d'un adénocarcinome mais sans aucune preuve de la tumeur primaire, lorsque l'identification de la tumeur primaire est impossible, un essai thérapeutique reste la seule méthode pour déterminer si les patients ont des tumeurs sensibles. Cependant, notre patient a refusé un traitement ultérieur, il a décédé 2 semaines plus tard et l'autopsie était refusée par sa famille.

## Conclusion

Les métastases choroïdiennes sont rarement révélatrices du cancer primitif; elles s'inscrivent souvent dans le cadre de néoplasie généralisée, elle sont de mauvais pronostic sur le plan fonctionnel et vital; la recherche du primitif doit être faite le plus rapidement possible afin de permettre une prise en charge thérapeutique, dominée surtout par une radiothérapie oculaire pour limiter l'extension et une chimiothérapie afin d'allonger la durée de survie des patients.
